# Evaluation and treatment of gastrointestinal bleeding in patients taking anticoagulants presenting to the emergency department

**DOI:** 10.1186/s12245-024-00649-7

**Published:** 2024-05-31

**Authors:** Adam J. Singer, Neena S. Abraham, Latha Ganti, W. Frank Peacock, Janaé Dark, Hajirah Ishaq, Ana Negrete, Brandon Mount, James Neuenschwander

**Affiliations:** 1 Stony Brook University, Stony Brook, NY USA; 2 Mayo Clinic, Rochester, MN USA; 3 Orlando College of Osteopathic Medicine, Winter Garden, FL USA; 4 Baylor College of Medicine, Houston, TX USA; 5 HCA Houston Healthcare, Clear Lake, TX USA; 6 Doctors Hospital, Columbus, OH USA; 7 Methodist University Hospital, Memphis, TN USA; 8 University of Toledo College of Medicine and Life Sciences, Toledo, OH USA; 9 Warren Alpert Medical School of Brown University, Providence, RI USA

**Keywords:** Gastrointestinal bleeding, Reversal of oral anticoagulant bleeding, Direct oral anticoagulants, Life threatening bleeding, Factor Xa inhibitors

## Abstract

This manuscript is a consensus document of an expert panel on the Evaluation and Treatment of Gastrointestinal Bleeding in Patients Taking Anticoagulants Presenting to the Emergency Department, sponsored by the American College of Emergency Physicians.

## Introduction

Anticoagulants are commonly used to treat or prevent thromboembolic events in patients with deep vein thrombosis, pulmonary embolism, and atrial fibrillation. While they significantly reduce morbidity and mortality from thromboembolic events, anticoagulants are associated with bleeding, which can be life-threatening. Compared to vitamin K-dependent oral anticoagulants (VKA’s) such as warfarin, direct-acting oral anticoagulants (DOACs) have a more rapid onset, a shorter duration of action, and a more predictable pharmacokinetic and pharmacodynamic profile, thus eliminating the need for routine therapeutic monitoring. DOACs also have fewer drug-drug and food-drug interactions than VKA. As a result, DOACs have overtaken warfarin as the most commonly used oral anticoagulant [1] and are recommended by evidence-based society guidelines for management of venous thromboembolism and prevention of stroke in non-valvular atrial fibrillation [2, 3]. While the risk of fatal bleeding in patients on DOACs is considerably less than in patients on VKA [4], it is estimated that major bleeding occurs in 4–6% of patients treated with oral factor Xa (FXa) inhibitors [5, 6]. 

When individual patient data from randomized clinical trials (RCT’s) of DOACs versus warfarin were analyzed collectively, standard-dose DOAC use resulted in a lower incidence of fatal and intracranial hemorrhage but no difference in major or gastrointestinal (GI) bleeding [7]. In contrast to results reported from RCTs under optimal trial conditions, real-world data from large observational studies demonstrated a higher risk of bleeding in patients taking DOAC, although still less than that associated with warfarin [8, 9]. In a population-based study comparing DOACs, investigators found apixaban had the most favorable GI safety profile and rivaroxaban the least favorable profile. In this analysis, GI bleeding events, among patients aged 75 years or older taking DOACs, increased with age. Apixaban had the most favorable GI safety profile among all age groups [9]. Of patients treated with FXa inhibitors and hospitalized for a major bleed, 56% had a major GI bleed [10]. The in-hospital mortality rate for patients with a DOAC-related GI bleed ranges from 2–7% [11–14]. Mortality may also be impacted by therapeutic intervention. A retrospective observational study of 3,030 patients that did not control for confounding variables found that mortality from GI bleeds ranged from 1% in patients treated with andexanet alfa to 4% in those treated with 4-factor prothrombin complex concentrate [12]. However, there are no randomized trials comparing these two agents head-to-head.

In 2013, visits to US EDs for adverse drug events were estimated to be 4 per 1000 individuals, with anticoagulants being the most common drug class implicated [15]. A study of trends in bleeding related to oral anticoagulants in US EDs between 2016 and 2020 demonstrated a 27.9% yearly increase in DOAC-related bleeds with an estimated rate of bleeding visits of 5.9 per 100 patients dispensed DOACs [16]. Of nearly 200,000 estimated DOAC-related bleeds in 2020, GI bleeding was the most commonly documented oral anti-coagulant related bleed [16]. 

The goal of this manuscript was to bring together clinicians from multiple disciplines (emergency medicine, gastroenterology, pharmacy) to summarize the evidence regarding the evaluation and treatment of patients taking anticoagulants presenting to the emergency department (ED) with GI bleed, and for the development of an American College of Emergency Physicians point-of-care tool for GI bleed management.

## Methodology

Topics were divided into subcommittee areas with committee members assigned to the various subcommittee topics. Topic reviews were the responsibility of each subcommittee to ensure comprehensive reviews, specifically including all available contemporary data within the last decade. After completion of their specific topics, each subcommittee presented their proposed statements, with their accompanying supportive documentation, to the overall committee. The overall committee then reviewed the presented topic proposals, contributing rebuttals and alternative proposals, which were then reconciled by overall consensus.

## History

The purpose of the history and physical examination is to determine the clinical significance of any GI bleed, as well as determine its etiology and location. Of all GI bleeds approximately 70% are upper and the rest are lower [17]. The presence of lightheadedness, dizziness, confusion, weakness, shortness of breath, chest pain, syncope, or near syncope may all suggest hemodynamic compromise and significant GI blood loss. Melena (black tarry stools) and hematemesis (bright red blood or coffee-ground emesis) suggest an upper GI source of bleeding, proximal to the ligament of Treitz, while hematochezia (bright red blood per rectum) generally suggests a lower GI bleeding source. Rarely, hematochezia, especially with hemodynamic instability, may be due to a brisk upper GI bleeding source. Mimics of GI bleeding should always be considered and ruled out. These include hemoptysis, vaginal bleeding, epistaxis, hematuria, and oral bleeding. Symptoms of underlying coagulopathy or bleeding disorder should also be sought including the presence of easy bruising, petechiae, or excessive bleeding from the gums.

A detailed prior history should include a history of GI bleeds, chronic alcohol use, gastric bypass surgery, repair of an abdominal aortic aneurysm, as well as the presence of renal or hepatic disease that may exacerbate bleeding. The results of recent prior colonoscopy or upper endoscopy may also be helpful. A history of peptic ulcer disease, esophageal varices, diverticulosis, arteriovenous malformation, inflammatory bowel disease, portal hypertension, Mallory-Weiss tear, or tumors of the GI tract may help identify the source of GI bleeding. A complete medication history should include the use of anticoagulants (including strength and timing of last dose), antiplatelet agents, nonsteroidal anti-inflammatory drugs, histamine type 2 receptor blockers, proton pump inhibitors, and steroids. In patients on oral anticoagulants, the use of food and drugs that interact with the anticoagulant should also be sought, especially those that increase the risk of excessive anticoagulation. The use of over-the-counter medications such as bismuth subsalicylate, vitamins, and supplements such as iron, may also help explain the presence of dark stool.

## Physical examination

A complete physical examination should start with an assessment of vital signs including heart rate and blood pressure. Hypotension and tachycardia suggest hypovolemic shock from significant blood loss. Other physical findings suggestive of poor perfusion include cool and clammy skin, weak pulses, delayed capillary refill, confusion, and poor urine output. The presence of pallor, scleral icterus, jaundice, ascites, bruising, and petechiae are also helpful in determining the underlying cause of bleeding. Signs of bleeding include melena, hematochezia, or hematemesis. Also be sure to exclude other sources of bleeding such as vaginal bleeding, hematuria, epistaxis, and oral bleeding. A shock index (SI), defined as the heart rate divided by systolic blood pressure, that exceeds 0.8–0.9, predicts a poorer prognosis, and a SI above 1 may indicate the need for massive transfusion [18, 19]. A modified SI, which is the ratio of heart rate to mean arterial pressure, greater than 1.3, may be more predictive of mortality than the SI [20]. 

## Testing and evaluation of GI bleeds

Laboratory testing in the presence of a GI bleed provides a snapshot of the patient’s current clinical status and assists in risk stratification. It is important to note that some tests may need to be repeated during the patient’s ED course. One of the most important blood tests in patients with GI bleeding is the type and screen. Crossmatching of blood should be completed once it is apparent that transfusion is likely to be required. A complete blood count (CBC) may suggest the need for transfusion, but clinicians should consider that hemoglobin and hematocrit (H/H) counts may lag behind clinical findings. A repeat of the H/H may be necessary if a brisk bleed is present. A low mean corpuscular volume (MCV) can indicate the presence of chronic iron deficiency anemia while a normal MCV is more common with an acute GI bleed. If the CBC results indicate the presence of chronic anemia, an anemia workup may be initiated. A basic metabolic panel (BMP) should be obtained. Elevated blood urea nitrogen (BUN) level may indicate blood in the alimentary canal, especially upper GI bleeds where a BUN/creatinine ratio is usually greater than 30:1 [21]. Reduced glomerular filtration rate may also prolong the anticoagulant effects of the DOACs especially dabigatran due to its dependence on renal elimination. Coagulation studies such as the prothrombin time (PT) and international normalized ratio (INR) are useful for assessing the intensity of warfarin therapy and hepatic synthetic function, and have the potential to detect exposure to FXa inhibitors based on the timing of the last dose of FXa inhibitor, the specific PT reagent and the specific FXa inhibitor [22]. As the INR is a mathematical calculation based upon reduction of vitamin K dependent clotting factors, it lacks meaning in patients treated with DOACs. Molecule-specific anti-Xa assays, when readily available, may be helpful when bleeding is due to low-molecular-weight heparin (LMWH) or FXa inhibitors such as apixaban (Eliquis ®), edoxaban (Savaysa ®), and rivaroxaban (Xarelto ®). The partial thromboplastin time (PTT) may be useful to indicate exposure to factor IIa inhibitors (e.g., dabigatran/Pradaxa®). A direct thrombin time can be helpful when assessing the residual anticoagulant effect of dabigatran. A normal thrombin time can exclude the presence of dabigatran while a dilute thrombin time can quantitate dabigatran concentrations. A hepatic function panel evaluates liver dysfunction, which could result in impaired coagulation factor synthesis. Cardiac biomarkers may be useful in certain clinical scenarios such as in patients with GI bleeding and cardiovascular disease and symptoms suggestive of acute ischemia such as chest pain. Serum iron concentration, total iron-binding capacity (TIBC), serum vitamin B-12, and serum folate should be ordered if there is suspicion of chronic anemia. Lactate and venous blood gas measurement can provide insight into perfusion status in patients with significant GI bleeding. Studies of blood viscoelasticity can evaluate platelet function and the ability to form and break down clots but are not clinically relevant or predictive of outcome in the setting of gastrointestinal bleeding [23]. A fibrinogen level should also be measured in patients with significant bleeding. A nasogastric aspirate is no longer required for the workup of a suspected upper GI bleeding. Upper endoscopy is the diagnostic test of choice in patients with upper GI bleeds, usually within the first 24 h [24]. Colonoscopy is generally not necessary since most lower GI bleeds will resolve spontaneously and adequate bowel preparation limits its urgent use. Computed tomography angiography (CTA) is the preferred diagnostic test of choice for unstable lower GI bleeds, while the utility of ultrasound is limited [25]. While a fecal occult blood test may help distinguish melena from iron it has no role in patients with significant GI bleeds [26]. 

## Treatment

Initial management of patients with GI bleeding includes aggressive resuscitation and stabilization of the hemodynamic status [27]. Patients should have rapid establishment of intravenous access by the placement of at least 2 large bore peripheral intravenous catheters. Central venous access or intraosseous access may be required when peripheral access is not possible. While awaiting blood products, repeated intravenous boluses of 250–500 ml of a warmed balanced crystalloid such as lactated Ringer’s solution should be given to maintain perfusion of vital organs such as the brain, heart, and kidneys. As with major trauma-associated bleeding, permissive hypotension should be considered while waiting for blood products. Packed red blood cells (PRBCs) should be given to unstable patients, especially with ongoing bleeding. Hemodynamically stable patients should be transfused with a target hemoglobin (Hgb) of 7 gm/dL [28]. A higher target Hgb (8–9 gm/dL) should be considered in those with active cardiovascular disease. Platelet transfusion should be considered if there is concurrent thrombocytopenia (platelet counts < 50,000/ml); however, platelets should not be given if thrombotic thrombocytopenic purpura (TTP) is suspected. Cryoprecipitate should be considered in patients with low fibrinogen levels (< 150–200 mg/dL). Patients requiring multiple transfusions of PRBCs (generally more than 2–4) should receive additional fresh frozen plasma and platelets at a 1:1:1 ratio to correct for any dilutional component of coagulopathy [29, 30]. Those who remain hemodynamically unstable, with evidence of hypoperfusion, may temporarily require vasopressors to support vital organ perfusion. Intravenous norepinephrine (Levophed®) may be started at doses of 0.02-1 mcg/kg/minute and titrated upward until reaching the target blood pressure.

In consultation with a gastroenterologist, endoscopic control of bleeding within 24 h should be considered. Unstable patients may also require interventional radiological procedures such as selective arterial embolization. Rarely, patients may require exploratory laparotomy for bleeding control.

## Reversal of oral anticoagulation

When to Reverse: The use of anticoagulant reversal agents should be reserved for patients presenting with a life-threatening GI hemorrhage, for example, hemodynamically unstable patients on vasopressors, a drop in Hgb of ≥ 5 g/dL, blood transfusion of ≥ 5 units of PRBCs, or patients at risk of death [31]. Patients with a major bleed based on International Society of Thrombosis and Hemostasis (ISTH) definitions (a drop of 2 g/dL in hemoglobin or transfusion of at least 2 units PRBC) [32] generally do not require acute reversal especially without ongoing blood loss and in patients with high risk of thrombosis. In these cases the oral anticoagulant should be held until the patient’s condition stabilizes.

How to Reverse: 4-factor prothrombin concentrate (4 F-PCC) [Kcentra®, Octaplex®] is indicated for life-threatening or uncontrolled bleeding in patients on an oral VKA and a supratherapeutic INR greater than 1.9 [33]. The exact dosage of 4 F-PCC is based on patient’s weight and pre-treatment INR as indicated in Table 1. Kcentra® is given at a rate of 0.12 mL/kg/min (∼ 3 units/kg/min) up to a maximum rate of 8.4 mL/min (∼ 210 units/min). A 5–10 mg intravenous dose of vitamin K is also indicated for these patients.

**Table 1:** Determination of 4 F-PCC dosage for reversal of coagulopathy

Pretreatment INR	2 to < 4	4 to 6	> 6
Dose of 4 F-PCC (Kcentra®) [units/kg]	25	35	50
Maximum dose [units]	2500	3500	5000

Andexanet alfa (Andexxa®) is indicated for life-threatening or uncontrolled bleeding in patients who have taken apixaban (Eliquis®) or rivaroxaban (Xarelto®) in the last 18–24 h [34]. The exact dose of Andexanet alfa is based on the specific Fxa inhibitor, dose, and timing of the last dose as indicated in Table 2. Andexanet alfa is given as an intravenous bolus over 15–30 min and followed by a continuous infusion over 2 h.

**Table 2:** Determination of dosing of Andexanet alfa

FXa Inhibitor	Strength of the last dose	Time from the last dose	
		< 8 h/ unknown	*≥* 8 h
Rivaroxaban	*≤* 10 mg	Low dose	Low dose
	> 10 mg/ unknown	High dose	
Apixaban	*≤* 5 mg	Low dose	Low dose
	> 5 mg/unknown	High dose	

If andexanet alfa (Andexxa®) is not available, 4 F-PCC (Kcentra®) should be considered at a dose of 25–50 units/kg not to exceed 5000 units [35]. Fixed doses of 4 F-PCC may help simplify administration and is recommended by some [36].

Idarucizumab (Praxabind®) is indicated for life-threatening or uncontrolled bleeding in patients recently taking dabigatran (Pradaxa®) [37]. The recommended dose of idarucizumab is 5 g IV if the last dose of dabigatran was within 24 h. Praxabind® (2 vials of 2.5 g/50 mL) is administered intravenously as two consecutive infusions over 5 to 10 min each or as a bolus injection. Prolonged dabigatran (Pradaxa®) effects may occur in the setting of renal insufficiency or failure. With all reversal agents there is a potential for an allergic reaction as well as venous or arterial thromboembolic events likely due to reversal of the anticoagulation in patients with an underlying clotting risk. Resumption of the anticoagulation as early as possible will help reduce this risk. The rates of thromboembolic rates have ranged from 6 to 10% after reversal of the anticoagulation with one of the three reversal agents [33, 34, 37].

The antidote for unfractionated and low molecular-weight heparin is protamine sulfate. However, it is only a partial reversal agent. If the patient has taken enoxaparin (Lovenox®) within 8 h, the dose of protamine sulfate is 1 mg per every 1 mg of enoxaparin (Lovenox®). If the patient has taken the enoxaparin (Lovenox®) more than 8 h but less than 12 h previously, the dose of protamine sulfate is 0.5 mg per 1 mg of Enoxaparin (Lovenox®). If the patient has taken the enoxaparin (Lovenox®) over 12 h previously, reversal may not be required. One milligram of protamine sulfate will neutralize approximately 100 units of unfractionated heparin (UFH). Since the half-life of UFH is approximately 90 min, the longer the time since the UFH is terminated, the less will be the effect of protamine sulfate. If a patient is on a heparin infusion, the amount administered over the previous two hours should be used to calculate how much protamine sulfate is to be given. In comparison, the half-life of protamine sulfate is just 7 min. It is reasonable to measure an activated partial thromboplastin time (aPTT) to assess the need of additional protamine sulfate. Adding a heparin anti Xa level may also be helpful in determining treatment.

## The role of surgical and interventional radiology

A CTA of the abdomen and pelvis may be considered as the initial diagnostic test in patients with ongoing hemodynamically significant hematochezia [38]. However, CTA is of low yield in patients with minor lower GI bleeding or those in whom bleeding has clinically subsided. Patients with a CTA demonstrating extravasation should be promptly referred to interventional radiology for transcatheter arteriography and possible embolization [39, 40]. For specialized centers with experience in performing endoscopic hemostasis, a colonoscopy can also be considered after a positive CTA [39]. 

There is a limited initial role for surgical evaluation in the setting of lower GI bleeding, and this option should only be considered after endoscopic or radiologic interventions have failed. In the setting of a known bleeding site and either recurrent or refractory bleeding, proceeding to targeted angiography with possible embolization is indicated despite endoscopic intervention.

Patients with overt upper GI bleeding, regardless of the predicted risk of further bleeding and death, should undergo upper endoscopy within 24 h of presentation [24, 31]. 

Patients with recurrent bleeding after endoscopic therapy of a bleeding ulcer should undergo repeat endoscopy. Surgery or interventional radiology with arterial embolization may be indicated for recurrent upper GI bleeding after failed repeated endoscopy and hemostatic therapy.

## Patient disposition

Disposition decisions should be guided by a combination of the patient’s clinical status, risk stratification scores, objective findings, and adherence to local standards of care. Use of the Glasgow-Blatchford score (GBS) [41] should guide clinicians on the appropriateness of discharge for patients presenting to the ED with an upper GI bleed, and the Oakland score [42] may provide direction regarding the discharge of ED patients with a lower GI bleed. A GBS less than or equal to 1 is the optimal cutoff for discharge of patients with acute upper GI bleeding. At this cut-off, roughly one in four patients can be safely discharged with a negative predictive value for adverse events of 100%.^31^ An Oakland score of 8–10 or lower can be used to identify patients with lower GI bleeds who are safe for discharge, with a sensitivity of adverse events of 96%.^32^ However, the Oakland score has not been studied in acute lower GI bleed and is not currently endorsed for routine use by the American College of Gastroenterology [18]. 

A referral for outpatient follow-up with a gastroenterologist should be provided upon discharge, with clear instructions on when to seek prompt medical attention. Admission to an observation unit may be appropriate in selected stable patients that would benefit from additional monitoring. Admission to a medical or surgical floor is appropriate for hemodynamically stable patients with clinical decision scores and other pertinent factors that indicate admission is warranted. Admission to a critical care unit is necessary for patients with brisk hemorrhage or hemodynamic instability especially those that require acute reversal for a life threatening bleed. Clinicians practicing in facilities without the appropriate specialist (either gastroenterology, surgery, or interventional radiology) should transfer the patient to a facility that can care for them.

## Data Availability

No datasets were generated or analysed during the current study.
